# How does the world appear to patients with multifocal intraocular lenses?: a mobile model eye experiment

**DOI:** 10.1186/s12886-020-01446-5

**Published:** 2020-05-06

**Authors:** Eun Chul Kim, Kyung-Sun Na, Hyun Seung Kim, Ho Sik Hwang

**Affiliations:** 1 Department of Ophthalmology, College of Medicine, The Catholic University of Korea, Seoul, Republic of Korea; 2 Department of Ophthalmology, Yeouido St. Mary’s Hospital, The Catholic University of Korea, 10, 63-ro, Yeongdeungpo-gu, Seoul, 07345 Republic of Korea

**Keywords:** Multifocal intraocular lens, Model eye, Mobile model eye, Simulation, Multifocal function, Halo

## Abstract

**Background:**

To show how the world appear to patients with multifocal intraocular lens (IOL) using a novel mobile model eye.

**Methods:**

The mobile model eye was composed of an artificial cornea, IOL, IOL chamber, and a camera. A monofocal IOL (Tecnis monofocal IOL) and two diffractive multifocal IOL (ReSTOR, Tecnis multifocal IOL) were used in the study. We went outside to take a picture of the scenery. At night, we stood on a road and took pictures to see how the traffic lights and headlights of cars looked. For an indoor analysis, we approached the Early Treatment Diabetic Retinopathy Study (ETDRS) chart to the model eye from a distance of 95 cm to check the multifocal function of the lenses. In the car, we took pictures of the street and a cell phone in turn to check the multifocal function of the lenses.

**Results:**

Two multifocal IOLs showed definite multifocal function. Far objects appeared either similarly clear or slightly hazier (depending on the IOL model) than those with the monofocal IOL. In the night vision, there was a mild or severe halo around light sources compared to those with the monofocal IOL.

**Conclusion:**

We believe that this mobile model eye can be used to evaluate how the real world appear to a patient with a multifocal IOL, to explain multifocal function of the IOLs, and possible complications in the patients, before performing a surgery.

## Background

The use of multifocal intraocular lenses (IOLs) in cataract surgeries has been increasing for years. Theoretically, patients with multifocal IOLs can clearly see both far and near objects. Then, how would the world actually look like to patients with multifocal IOLs? Can the patients clearly see objects that are both far and near? Do far objects look less clear with a multifocal IOL compared to those with a monofocal IOL? Do patients see a halo or a starburst around the light while driving at night?

We cannot know the exact answers to the abovementioned questions unless we implant the multifocal IOLs in our own eye. Even if we implant these lenses in our eyes and know how the world appear, it is very difficult to objectively convey it to another person.

Although there are many reports that show near, intermediate, and long-term visual acuity in patients after implantation of multifocal IOLs and also question whether the vision is blurred at night, especially halos around lights, particularly street lights and oncoming traffic lights [1–12]. These are all subjective tests that ask the patients. There are some studies that have used resolution targets and an optical bench to evaluate the use of multifocal IOLs [13–16]. However, patients and even clinicians cannot understand the results of the optical bench test. Moreover, these studies are limited in terms of describing how patients actually see the world with the help of such simple targets.

In this study, we designed a compact mobile model eye and implanted a monofocal IOL and two diffractive multifocal IOLs. Subsequently, we photographed the real world (far objects, near objects, traffic light at night, cell phone, and other objects) to examine how the world appear to patients with multifocal IOLs.

## Methods

**Fig. 1:**
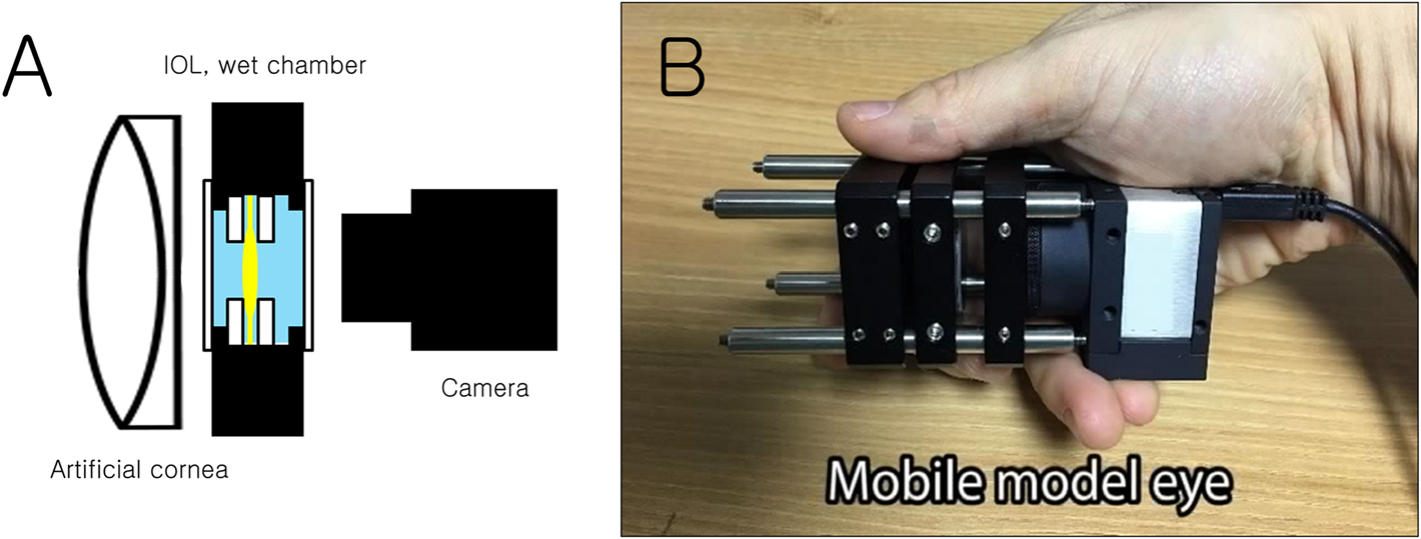
Schematic diagram (**a**) and photo of the mobile model eye (**b**). The model eye was composed of an artificial cornea, intraocoular lens (IOL) chamber, and a camera (This figure was created by Ho Sik Hwang)

The model eye was composed of an artificial cornea, IOL chamber, and a camera (Fig. 1). We used an achromatic lens (focal length: 150 mm) as an artificial cornea. We used an achromatic lens with a focal length of 150 mm as an artificial cornea. If we use an achromatic lens with the focal length of 23.3 mm (43 D), it will be similar to the human cornea. But, for our model eye, if the focal length is shorter than 150 mm, even if the IOL chamber contacts camera (the distance between the IOL chamber and the camera is 0), the distant image target is focused in front of the sensor of camera. So, we chose an achromatic lens with the focal length of 150 mm as an artificial cornea. IOL was placed in the distilled water-filled chamber. The chamber had two parallel N-BK7 windows. The IOL mount had 3.8-mm-sized clear aperture. The IOL centration was confirmed by a dissection microscope after mounting the IOL. Finally, a color complementary metal–oxide–semiconductor (CMOS) camera (resolution 1280 × 1024) was connected. The artificial cornea, IOL chamber, and camera were positioned within the 30-mm cage system with four rigid steel rods, thereby eliminating the need of an additional alignment. We need only the focusing procedure.

A monofocal IOL (Tecnis monofocal ZCB00, Johnson & Johnson vision, Santa Ana, CA)(+ 20.0 diopter [D]) and two diffractive multifocal IOLs (AcrySof® IQ ReSTOR® (Alcon, Fort Worth, TX) (base power + 20.0 D, add power + 2.50 D) and Tecnis multifocal ZKB00 IOL (Johnson & Johnson vision) (base power + 20.0 D, add power + 2.75 D)) were used in the study. The purpose of this study is not to report the characteristics of a specific lens, but to report the applicability of the mobile model eye.

The artificial cornea and the IOL chamber were tightly attached (the distance between the artificial cornea and the IOL chamber is 0). Then the distance from the posterior surface of the artificial cornea to the center of the IOL optic was 4.7 mm and kept constant. The CMOS camera was connected to a laptop which allowed us to check the image.

**Fig. 2:**
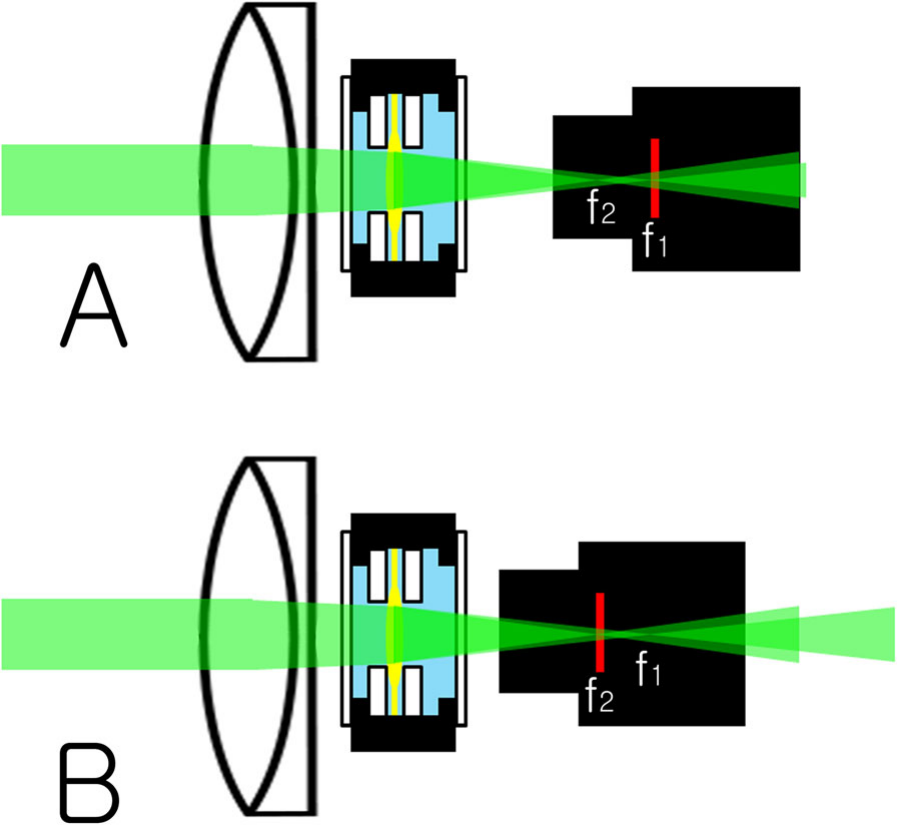
Focusing of model eye. For a distant image target (more than 6 m away from the model eye), the distance between the IOL chamber and the camera was adjusted so that the image on the computer monitor could be seen most clearly. In the case of bifocal multifocal IOL, this happens at two locations. The first is the position where the distant image target is focused at the sensor of the CMOS camera by the base power of the IOL (**a**). At the same time, the distant image target is also focused in front of the sensor by the add power of the IOL. The second is the position where the distant image target is focused at the sensor by the add power of IOL (**b**). At the same time, the distant image target is also focused behind the sensor by the base power of the IOL. In this experiment, the artificial cornea, the IOL chamber, and the camera were fixed at the first position. f1: focus by base power of the IOL f2: focus by add power of the IOL (This figure was created by Ho Sik Hwang)

For a distant image target (more than 6 m away from the model eye), the distance between the IOL chamber and the camera was adjusted so that the image on the computer monitor could be seen most clearly (The distance between the posterior surface of the artificial cornea and the sensor of the camera was 41.6 mm). In the case of bifocal multifocal IOL, this happens at two locations (Fig. 2). The first is the position where the distant image target is focused at the sensor of the CMOS camera by the base power of the IOL (The distance between the posterior surface of the artificial cornea and the sensor of the camera was 41.6 mm). At the same time, the distant image target is also focused in front of the sensor by the add power of the IOL. The second is the position where the distant image target is focused at the sensor by the add power of IOL. At the same time, the distant image target is also focused behind the sensor by the base power of the IOL. In this experiment, the artificial cornea, the IOL chamber, and the camera were fixed at the first position (The distance between the posterior surface of the artificial cornea and the sensor of the camera was 41.6 mm). Then, a black tape was used to shield the space between the IOL mount and the camera from ambient light.

**Fig. 3:**
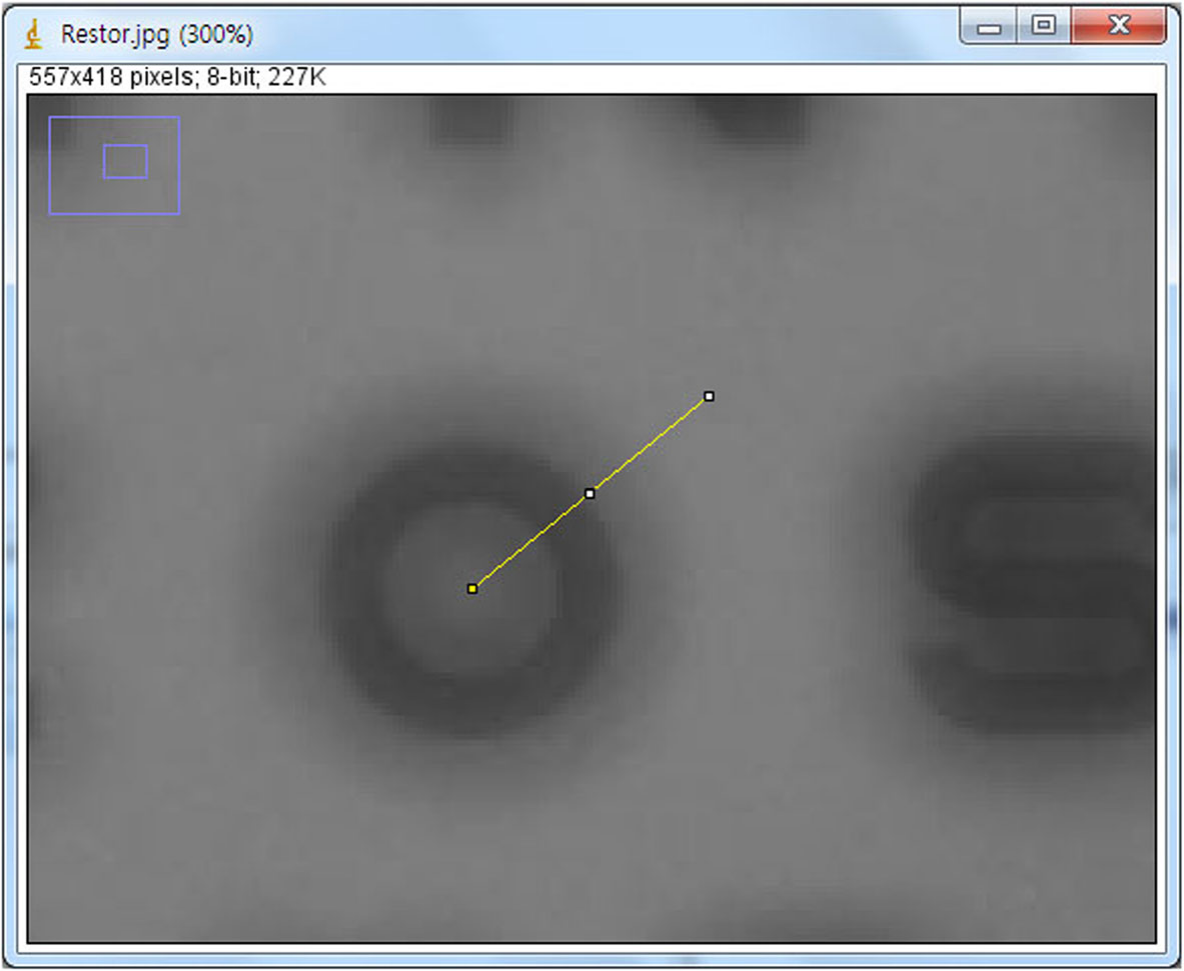
Quantitative analysis of ETDRS chart image. For quantitative analysis, we analyzed the ETDRS chart image at 40 cm from the mobile mode eye for Tecnis monofocal IOL and ReSTOR and at 36 cm for Tecnis multifocal IOL. Color image were converted to greyscale image. We measured pixel intensity (GreyValue) from the center of character ‘O’ in ETDRS chart using Image J (https://imagej.nih.gov/) (length: 500 pixels) (This figure was created by Ho Sik Hwang)

We held the laptop in one hand and the model eye in the other and went outside to record video of the scenery (camera setting: exposure time auto, white balance auto, gain auto). At night, we stood on a road and took pictures to see how the traffic lights, headlights, and tail lights of cars looked. For an indoor analysis, we approached the Early Treatment Diabetic Retinopathy Study (ETDRS) chart (ETDRS 2000 Series chart “2”(Precision Vision, La Salle, IL) to the model eye from a distance of 95 cm to check the multifocal function of the lenses. For quantitative analysis, we analyzed the ETDRS chart images at 40 cm from the mobile mode eye for Tecnis monofocal IOL and ReSTOR and at 36 cm for Tecnis multifocal IOL. Color image were converted to greyscale image. We measured intensities of pixels (GreyValue) on the reference line (length: 500 pixels) from the center of character ‘O’ in ETDRS chart using Image J (https://imagej.nih.gov/) (Fig. 3). We calculated contrast (%) as 100% * (Imax-Imin)/(Imax+Imin) (Imax: Maxium of intensity, Imin: Minimum of intensity). In the car, we took pictures of the street and a cell phone in turn to check the multifocal function of the lenses.

## Results

### Outdoor

**Fig. 4:**
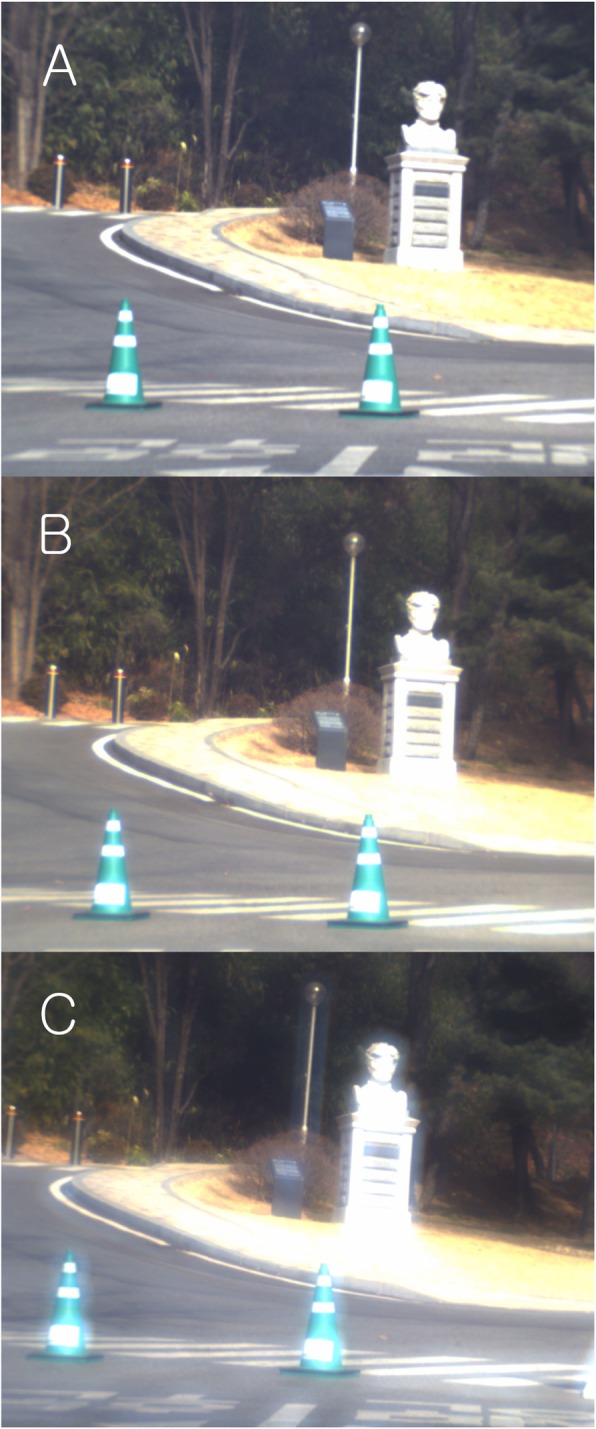
Outdoor. A university building was photographed. A stone statue, traffic cones, streetlights, trees, and windows of the building at a distance were clearly visible with the monofocal IOLs (**a**). RESTOR showed similar observation as the monofocal IOLs (**b**). However, with the Tecnis multifocal IOL, the images were slightly hazier than those with the monofocal IOL. In particular, there were halos around bright objects such as the stone statue, traffic cones, metal pole of the streetlight, and sign boards (**c**) (This figure was created by Ho Sik Hwang)

A university building was photographed. A stone statue, traffic cones, streetlights, trees, and windows of the building at a distance were clearly visible with the monofocal IOLs (Fig. 4a). ReSTOR showed similar observation as the monofocal IOLs (Fig. 4b).

However, with Tecnis multifocal IOL, the images were slightly hazier than those with the monofocal IOL. In particular, there were halos around bright objects such as the stone statue, traffic cones, metal pole of the streetlight, and sign boards (Fig. 4c).

### Night vision

**Fig. 5:**
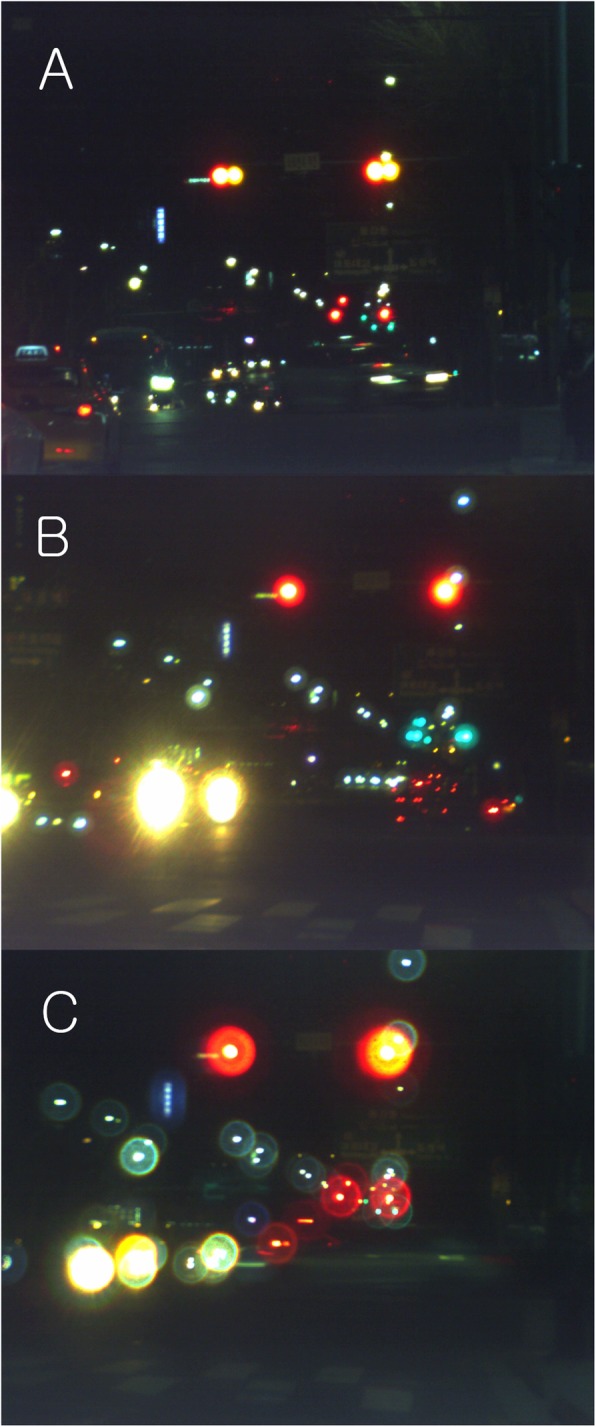
Night vision. Traffic lights, headlights, and tail lights of cars looked slightly blurrier with monofocal IOLs than the naked eye (**a**). RESTOR showed slight halos around each light source than with the monofocal IOL (**b**). With Tecnis multifocal IOL, halos were more severe than with the monofocal IOL (**c**) (This figure was created by Ho Sik Hwang)

Traffic lights, headlights, and tail lights of cars looked slightly blurrier with monofocal IOLs than the naked eye (Fig. 5a). ReSTOR showed slight halos around each light source than with the monofocal IOL (Fig. 5b). With Tecnis multifocal IOL, halos were more severe than with the monofocal IOL (Fig. 5c).

### Near target

**Fig. 6:**
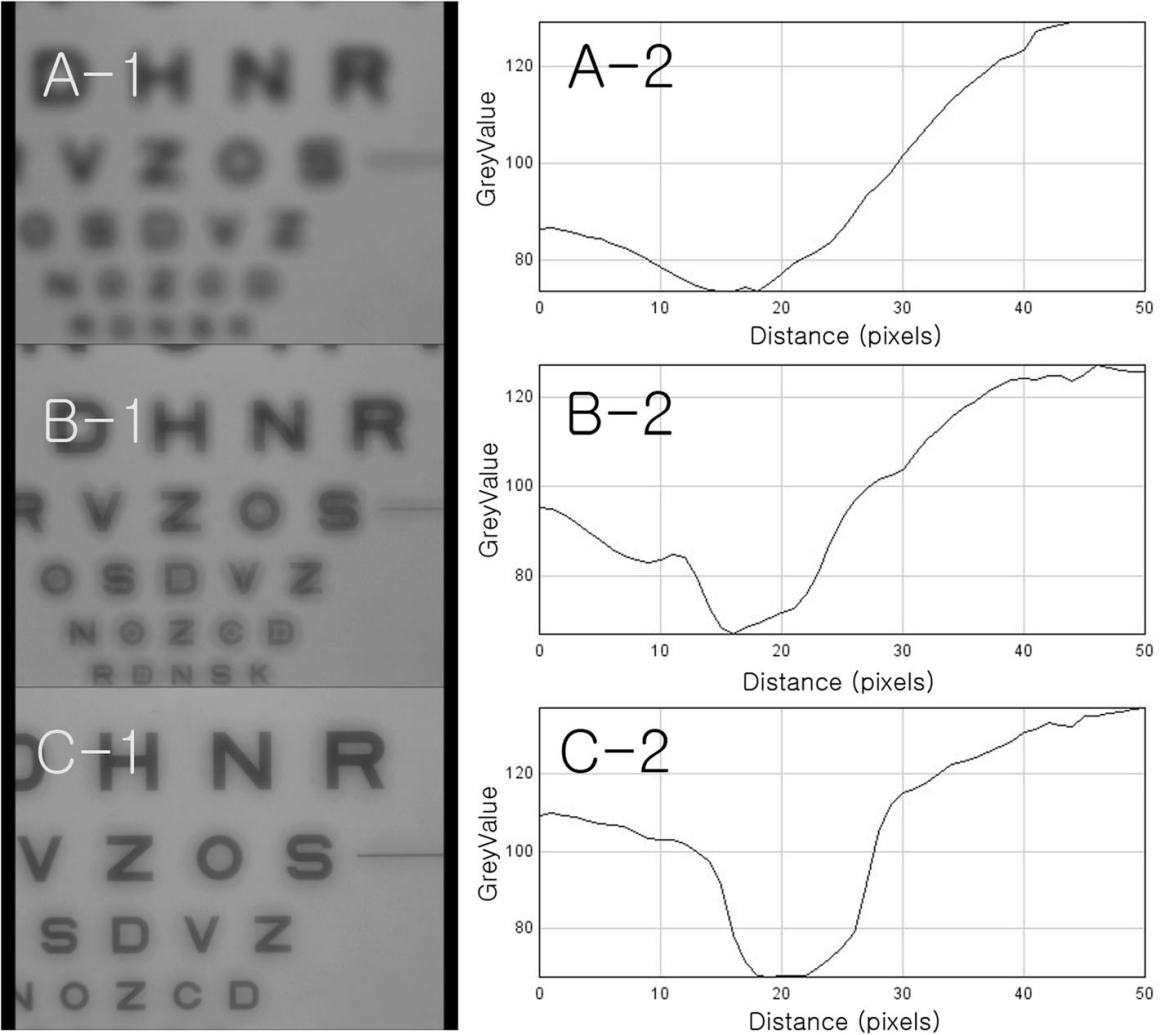
Near target. With the monofocal IOL, the ETDRS chart continued to blur as it approached from a distance of 95 cm (**A-1**). ETDRS chart image at 40 cm from the model eye use for quantitative analysis. Intensity profile from the center of character ‘O’ in the ETDRS chart showed that the intensity of pixels decreased until the measurement point reached the dark ink line of character ‘O’. And it increased again as the measurement point moved out. But, the slope was not steep (**A-2**). The contrast of character ‘O’ was 27.3%. With ReSTOR, the ETDRS chart blurred as it got closer, but became clear again at a distance of around 40 cm. However, halos were observed around the letters (**B-1**). Intensity profile showed steeper slope around the dark ink line of character ‘O’ than monofocal IOL (**B-2**). The contrast of character ‘O’ was 30.4%. With Tecnis multifocal IOL, the ETDRS chart blurred as it got closer, but became clear at a distance of around 36 cm. It was clearer than with ReSTOR, though with faint halos around the letters (**C-1**). Intensity profile showed steeper slope around the dark ink line of character ‘O’ than ReSTOR (**C-2**). The contrast of character ‘O’ was 33.9% (This figure was created by Ho Sik Hwang)

With the monofocal IOL, the ETDRS chart continued to blur as it approached from a distance of 95 cm (Fig. 6A-1). ETDRS chart image at 40 cm from the model eye use for quantitative analysis. Intensity profile from the center of character ‘O’ in the ETDRS chart showed that the intensity of pixels decreased until the measurement point reached the dark ink line of character ‘O’. And it increased again as the measurement point moved out. But, the slope was not very steep (Fig. 6A-2). The contrast of character ‘O’ was 27.3%. With ReSTOR, the ETDRS chart blurred as it got closer, but became clear again at a distance of around 40 cm. However, halos were observed around the letters (Fig. 6B-1). Intensity profile showed steeper slope around the dark ink line of character ‘O’ than monofocal IOL (Fig. 6B-2). The contrast of character ‘O’ was 30.4%. With Tecnis multifocal IOL, the ETDRS chart blurred as it got closer, but became clear at a distance of around 36 cm. It was clearer than with ReSTOR, though with faint halos around the letters (Fig. 6C-1). Intensity profile showed steeper slope around the dark ink line of character ‘O’ than ReSTOR (Fig. 6C-2). The contrast of character ‘O’ was 33.9%.

### Smartphone

**Fig. 7:**
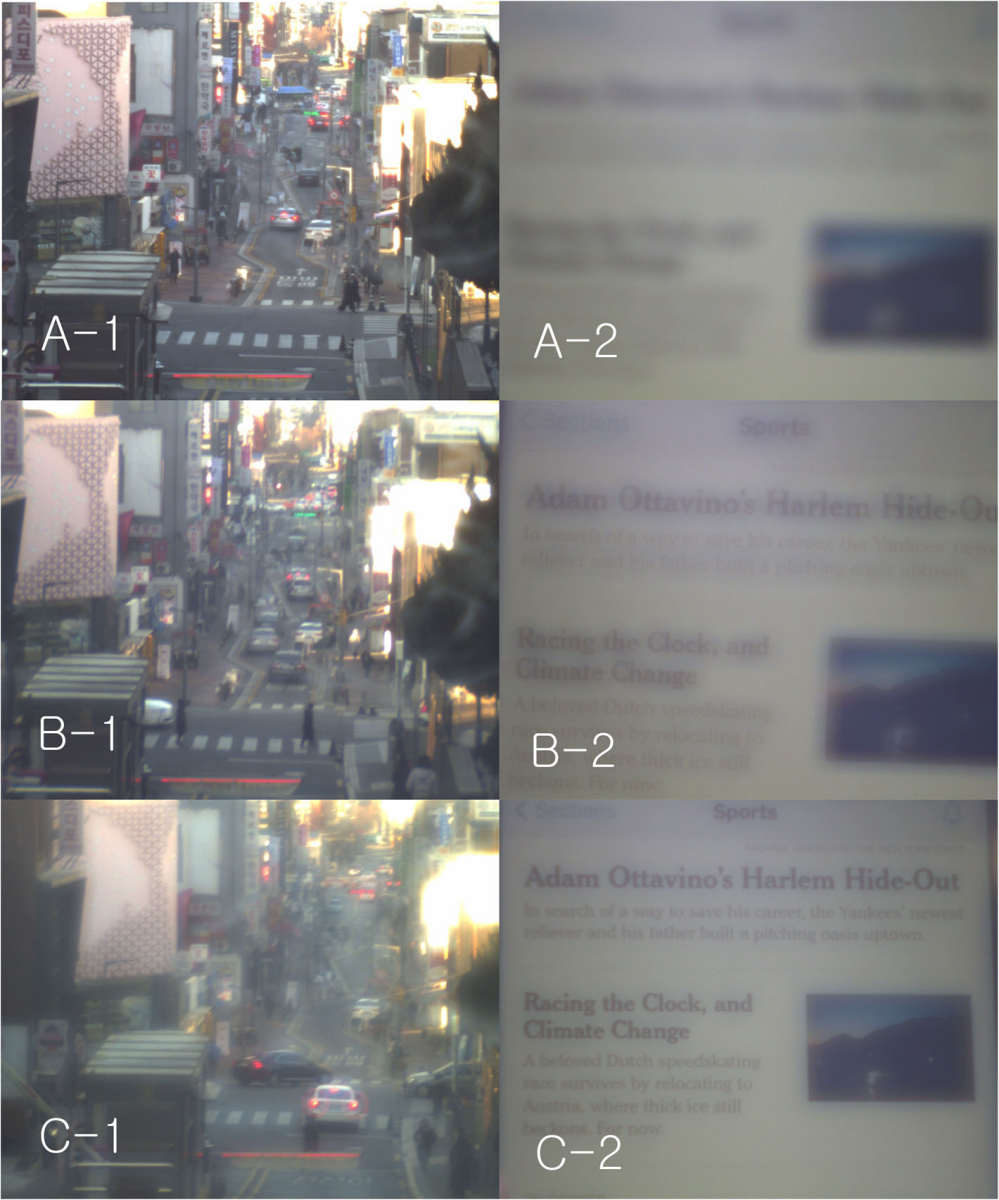
Street and smartphone. With the monofocal IOL, the letters were so blurry that they could not be read from a distance of about 40 cm (**A-2**). With ReSTOR, the letters could be read from a distance of about 40 cm. But, the letters were not very clear because of the halo (**B-2**). With Tecnis multifocal IOL, the letters could be read from a distance of about 40 cm. Moreover, they were clearer than those with ReSTOR because the ghost images around these letters were dim (**C-2**) (This figure was created by Ho Sik Hwang)

With the monofocal IOL, the letters were so blurry that they could not be read from a distance of about 40 cm (Fig. 7a). With ReSTOR, the letters could be read from a distance of about 40 cm. But, the letters were not very clear because of the halo (Fig. 7b). With Tecnis multifocal IOL, the letters could be read from a distance of about 40 cm. Moreover, they were clearer than those with ReSTOR because the ghost images around these letters were dim (Fig. 7c).

## Discussion

In this study, we designed a compact mobile model eye and implanted a monofocal IOL and two diffractive multifocal IOLs. We photographed the real world (far objects, near objects, traffic light at night, cell phone, and other objects) to examine how the world appear to patients with multifocal IOLs.

Using this mobile model eye, we found that with a multifocal IOL, first, two multifocal IOLs showed definite multifocal function. Second, far objects appeared either similarly clear or slightly hazier (depending on the IOL model) than those with the monofocal IOL. Third, in the night vision, there was a mild or severe halo (depending on the IOL model) around light sources compared to those with the monofocal IOL.

**Fig. 8:**
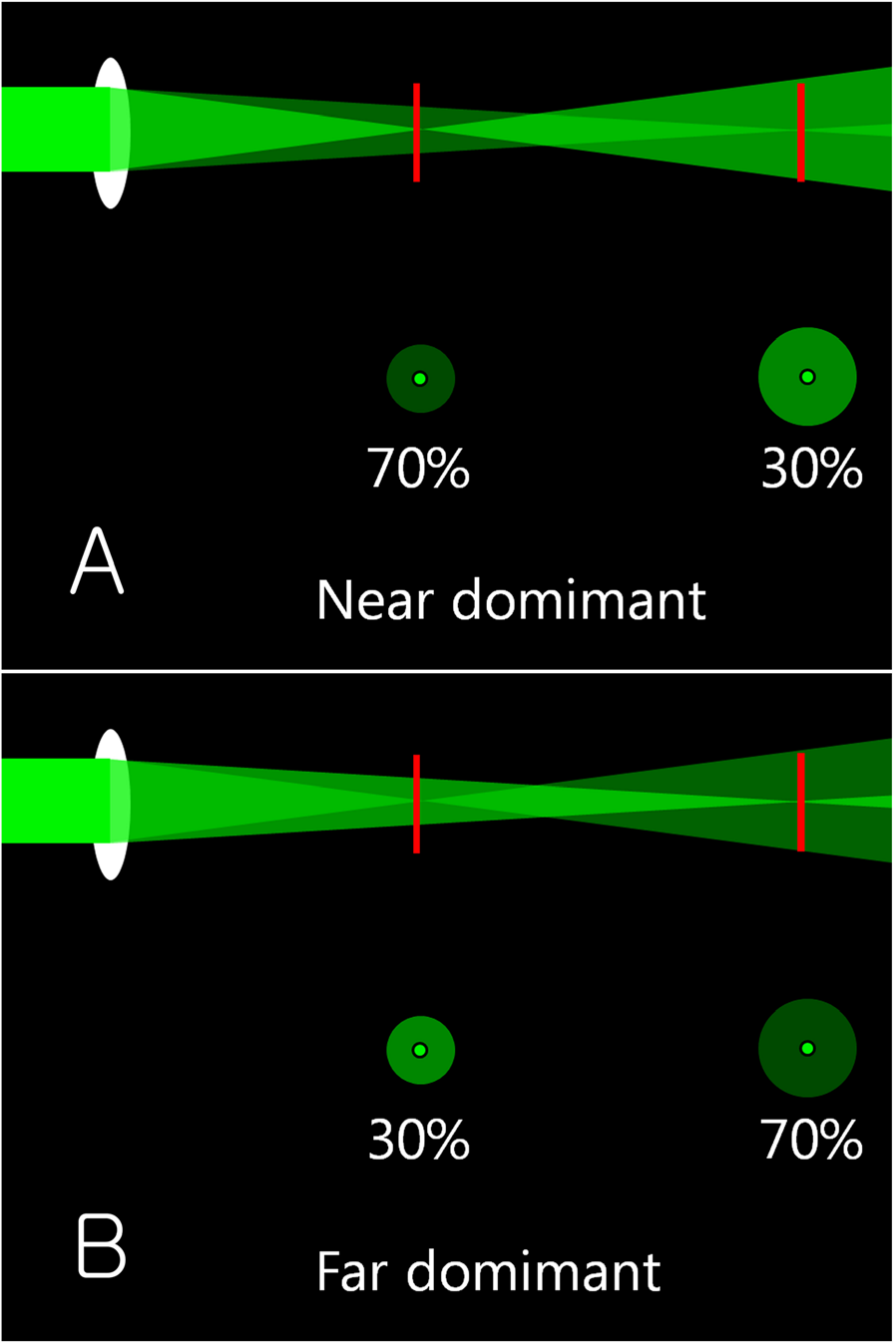
Light energy distribution at near and far distances. If 30% of the light energy goes to the near focus and 70% of the light energy goes to the far focus, the halo will be strong at the near distance, but will be weak at the far distance (**a**). Conversely, if 70% of the light energy goes to the near focus and 30% goes to the far focus, the halo will be weak at the near distance, but will be strong at the far distance (**b**) (This figure was created by Ho Sik Hwang)

Interestingly, with ReSTOR, halo was definite around the letters at a near distance, while it was not significant at a far distance in the daytime. On the contrary, with Tecnis multifocal IOL, halo was faint around the letters at the near distance, while it was significant at a far distance. This difference may occur because of the different IOL designs that differently distribute the light energy at near and far distances. If 30% of the light energy goes to the near focus and 70% of the light energy goes to the far focus, the halo will be strong at the near distance, but will be weak at the far distance (Fig. 8a). Conversely, if 70% of the light energy goes to the near focus and 30% goes to the far focus, the halo will be weak at the near distance, but will be strong at the far distance (Fig. 8b). Therefore, it is recommended to choose a multifocal IOL depending on whether the near vision or the distant vision is more important to the patient.

In the night vision, there was a mild or severe halo (depending on the IOL model) around light sources compared to those with the monofocal IOLs (Fig. 5b,c). Therefore, there is a concern that a patient with a multifocal IOL implant has a great inconvenience while driving at night. Even during the day, bright objects caused halo (or ghost image) around them (Fig. 4c). Patients may not feel such a severe halo because of neural adaptation in the brain [17]. Numerous research studies show that motivated patients were satisfied with the multifocal IOLs and mild photopic phenomena decreases as time goes by [1–12]. But, the formation of halos seems to be inevitable according to the principle of multifocal IOL.

Choi et al. tried to simulate night driving of patients with multifocal IOLs [18]. Their model eye was attached to a Nikon D70 digital SLR camera (Nikon Corp, Tokyo, Japan) to photograph a night driving scene with each IOL. The night driving scene showed that ReSTOR lens has less stray light artifacts compared to the Tecnis ZM900. This is similar to our results. But, we took pictures of not only night vision but also day scene, near objects and cell phones using mobile model eye.

To quantify photic phenomena of multifocal IOL, Giers et al. used a computer simulator software (Halo & Glare Simulator, EyelandDesign Network GmbH, Vreden, Germany) [19]. Patients select from different kinds of halos and glare and then adjust for size and intensity by moving a slide bar with simultaneous visual representation on the screen. But, this software does not simulate the photic phenomena using a model eye before cataract surgeries but was used to assess photic phenomena of patients with multifocal IOLs after cataract surgeries.

Radhakrishnan et al. tried to simulate the vision of multifocal IOL patients [20]. The visual simulator consisted of two rectilinear optical channels, each one with a tunable lens (EL-10-30-C, Optotune Inc., Switzerland), two projection lenses and an erecting prism. But, their simulator did not use real IOLs for simulation.

**Fig. 9:**
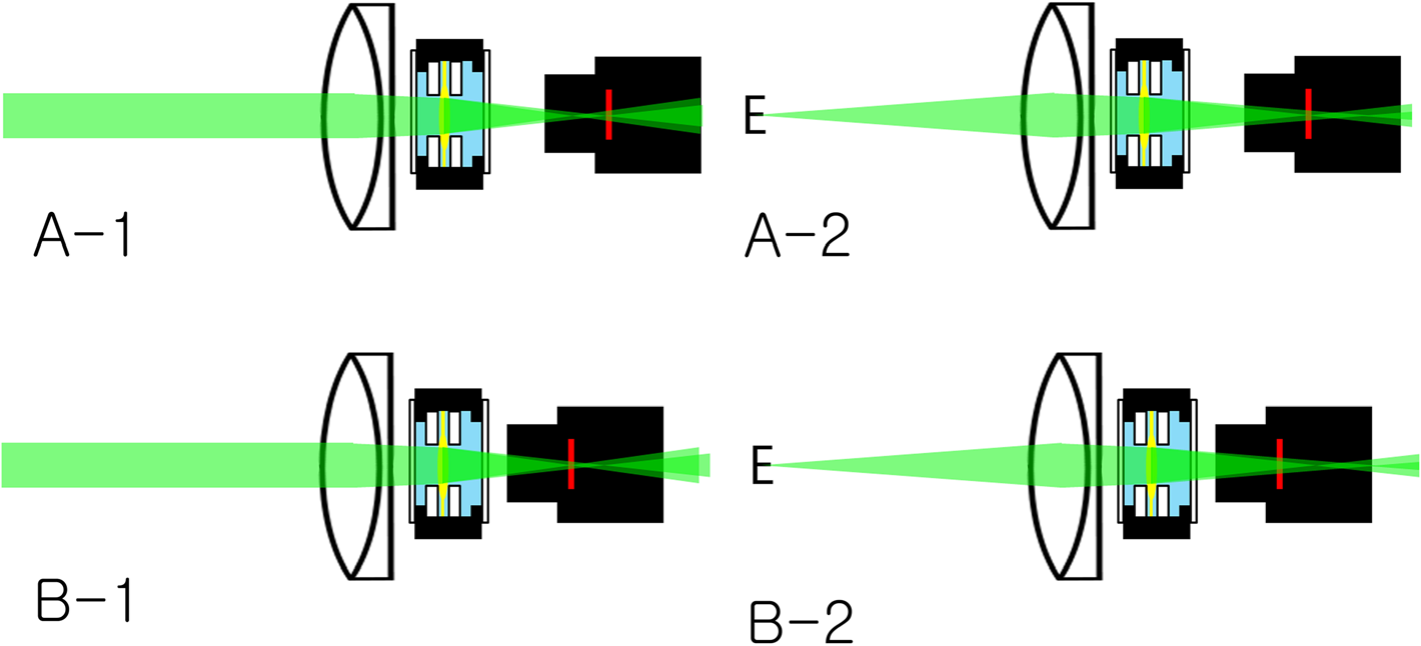
Focusing of model eye. For bifocal IOL, the distant image target by the base power of the IOL should be focused at the sensor of the CMOS camera and the distant image target by the add power of the IOL be focused in front of the sensor (**A-1**). In this case, the near image target is focused at the sensor by add power, so the lens shows bifocal function (**A-2**). If the distant image target by the add power is focused at the sensor (**B-1**), we cannot find the multifocal function of the multifocal lens (**B-2**) (This figure was created by Ho Sik Hwang)

Focusing is very important at the beginning of the experiment. For bifocal IOL, the distant image target by the base power of the IOL should be focused at the sensor of the CMOS camera and the distant image target by the add power of the IOL be focused in front of the sensor by the add power of the IOL (Fig. 9a). In this case, the near image target is focused at the sensor by add power, so the lens shows bifocal function. If the distant image target by the add power is focused at the sensor, we cannot find the multifocal function of the multifocal lens (Fig. 9b).

This study has some limitations. First, we took pictures of street to shows how the world appear to patients with multifocal IOLs using the mobile model eye. But, the street scenes continuously vary unlike a standard resolution target in a laboratory. A standard resolution target would be better to compare the function of different lenses. Second, we used an achromatic lens with a focal length of 150 mm as an artificial cornea. In order to use an achromatic lens with the focal length 23.3 mm (43 D), we have to use the objective lens and it makes the model eye bigger and results in imperfect alignment. Third, we used an achromatic lens as an artificial cornea. But, this is not identical to real human cornea because human cornea has spherical aberration and human eye has chromatic aberration [16, 18]. Fourth, in this study, we used a 3.8-mm-sized pupil. Using IOL mounts with different clear aperture sizes, we can also see the effect of the pupil size. Or we can place iris diaphragms in front of the IOL mount for a different pupil size. Fifth, we used automatic camera setting (exposure time auto, white balance auto) for video recording. We didn’t use contrast change of the images. But, it would be better to use identical setting for comparison of different IOLs. So, we don’t think that this mobile model eye replicates exactly what the patient will see.

## Conclusions

This is the first report that shows how the world appear to patients with multifocal IOLs using the mobile model eye. We believe that this mobile model eye can be used to evaluate how the real world appear to a patient with a multifocal IOL, to explain multifocal function of the IOLs, and possible complications in the patients, before performing a surgery. Patients can choose monofocal or multifocal IOLs also the specific lens among multifocal IOLs using mobile model eye simulation. We will use an artificial cornea close to real human cornea in next experiment.


**Additional file 1**: **Video 1.** Outdoor. A university building was photographed. A stone statue, traffic cones, streetlights, trees, and windows of the building at a distance were clearly visible with the monofocal IOLs. ReSTOR showed similar observation as the monofocal IOLs. However, with Tecnis multifocal IOL, the images were slightly hazier than those with the monofocal IOL. In particular, there were halos around bright objects such as the stone statue, traffic cones, metal pole of the streetlight, and sign boards (Multifocal IOL A: ReSTOR, multifocal IOL B: Tecnis multifocal IOL) (This video was created by Ho Sik Hwang).



**Additional file 2**: **Video 2.** Night vision. Traffic lights, headlights, and tail lights of cars looked slightly blurrier with monofocal IOLs than the naked eye. ReSTOR showed slight halos around each light source than with the monofocal IOL. With Tecnis multifocal IOL, halos were more severe than with the monofocal IOL (Multifocal IOL A: ReSTOR, multifocal IOL B: Tecnis multifocal IOL) (This video was created by Ho Sik Hwang).



**Additional file 3**: **Video 3.** Near target. With the monofocal IOL, the ETDRS chart continued to blur as it approached from a distance of 90 cm. With ReSTOR, the ETDRS chart blurred as it got closer, but became clear again at a distance of around 40 cm. However, halos were observed around the letters. With Tecnis multifocal IOL, the ETDRS chart blurred as it got closer, but became clear at a distance of around 40 cm. It was clearer than with ReSTOR, though with faint halos around the letters (Multifocal IOL A: ReSTOR, multifocal IOL B: Tecnis multifocal IOL) (This video was created by Ho Sik Hwang).



**Additional file 4**: **Video 4.** Street and smartphone. With the monofocal IOL, the letters were so blurry that they could not be read from a distance of about 40 cm. With ReSTOR, the letters could be read from a distance of about 40 cm. But, the letters were not very clear because of the halo. With Tecnis multifocal IOL, the letters could be read from a distance of about 40 cm. Moreover, they were clearer than those with ReSTOR because the ghost images around these letters were dim (Multifocal IOL A: ReSTOR, multifocal IOL B: Tecnis multifocal IOL) (This video was created by Ho Sik Hwang).


## Data Availability

For more information of the study, please contact the corresponding author.

## Abbreviations

IOL: Intraocular lens
CMOS: Complementary metal–oxide–semiconductor
D: Diopter
ETDRS: Early Treatment Diabetic Retinopathy Study
Imax: Maxium of intensity
Imin: Minimum of intensity
SLR: Single-lens reflex camera
